# Attenuated Post-Movement Beta Rebound Associated With Schizotypal Features in Healthy People

**DOI:** 10.1093/schbul/sby117

**Published:** 2018-09-18

**Authors:** Benjamin A E Hunt, Elizabeth B Liddle, Lauren E Gascoyne, Lorenzo Magazzini, Bethany C Routley, Krish D Singh, Peter G Morris, Matthew J Brookes, Peter F Liddle

**Affiliations:** 1 Diagnostic Imaging, The Hospital for Sick Children, Toronto, ON, Canada; 2 Program in Neurosciences and Mental Health, The Hospital for Sick Children Research Institute, Toronto, ON, Canada; 3 The Sir Peter Mansfield Imaging Centre, University of Nottingham, Nottingham, UK; 4 The Institute for Mental Health, University of Nottingham, Nottingham, UK; 5 Cardiff University Brain Research Imaging Centre, School of Psychology, Cardiff University, Cardiff, UK

**Keywords:** magnetoencephalography, schizotypy, schizophrenia, individual differences, schizotypal personality disorder, psychosis

## Abstract

**Introduction:**

Schizophrenia and schizotypal personality disorder (SPD) lie on a single spectrum of mental illness and converging evidence suggests similarities in the etiology of the 2 conditions. However, schizotypy is a heterogeneous facet of personality in the healthy population and so may be seen as a bridge between health and mental illness. Neural evidence for such a continuity would have implications for the characterization and treatment of schizophrenia. Based on our previous work identifying a relationship between symptomology in schizophrenia and abnormal movement-induced electrophysiological response (the post-movement beta rebound [PMBR]), we predicted that if subclinical schizotypy arises from similar neural mechanisms to schizophrenia, schizotypy in healthy individuals would be associated with reduced PMBR.

**Methods:**

One-hundred sixteen participants completed a visuomotor task while their neural activity was recorded by magnetoencephalography. Partial correlations were computed between a measure of PMBR extracted from left primary motor cortex and scores on the Schizotypal Personality Questionnaire (SPQ), a self-report measure of schizotypal personality. Correlations between PMBR and SPQ factor scores measuring cognitive-perceptual, interpersonal and disorganization dimensions of schizotypy were also computed. Effects of site, age, and sex were controlled for.

**Results:**

We found a significant negative correlation between total SPQ score and PMBR. This was most strongly mediated by variance shared between interpersonal and disorganization factor scores.

**Conclusion:**

These findings indicate a continuum of neural deficit between schizotypy and schizophrenia, with diminution of PMBR, previously reported in schizophrenia, also measurable in individuals with schizotypal features, particularly disorganization and impaired interpersonal relations.

## Introduction

The Diagnostic and Statistical Manual (DSM-5)^1^ now recognizes a gradient in the severity of psychotic disorders. The spectrum extends to schizotypal personality disorder (SPD), involving distortions of reality, odd speech and behavior, and difficulty relating to others, but not reaching the severity threshold for diagnosis of a psychotic disorder. Growing evidence indicates that the patho- physiological mechanisms underlying SPD are similar to those occurring in schizophrenia, though less severe.^2^

The concept of a schizotypal continuum has deep historical roots. Claridge^3,4^ proposed a continuum of schizo typal features extending across the general population and reaching its most severe form in schizophrenia itself. In cases not reaching the severity required for a diagnosis of schizophrenia, but in whom the traits produce substantial impairments in self and interpersonal functioning, a diagnosis of SPD might be justified. Chapman and Chapman^5^ noted that non-patients report isolated psychotic experiences and attenuated versions of psychotic experiences. Meehl^6^ introduced the concept of schizotaxia, which he proposed was a genetically determined neural integrative defect underlying schizotypy. He suggested that subtle neurological and physiological disorders are closer to the genes underlying schizophrenia than are social, or high-level cognitive processes. In particular, there is evidence of problems with fine motor control associated with schizotypal features in nonclinical samples^7^and in cases of SPD.^8,9^

Factor analyses of clinical features have revealed multiple dimensions in schizophrenia, 3 of which might be considered characteristic of the illness: reality distortion (delusions and hallucinations), disorganization (odd speech, affect and behavior), and psychomotor poverty (diminished speech, affect and behavior).^10^ A similar 3-factor structure has emerged from factor analyses of schizotypy.^11,12^ An “unusual experience,” or “cognitive-perceptual” dimension includes distortions of reality such as perceptual aberrations and unusual ideas; a “disorganization” dimension comprises odd speech and behavior, while a “negative” or “interpersonal” dimension includes loss of normal emotional, physical, and social function. In schizophrenia, persisting disorganization and psychomotor poverty are strongly associated with impaired cognitive, occupational and social function.^10,13^ Similarly, cases with disorganized and/or negative schizotypy demonstrate reduced well-being compared with individuals whose profile is characterized by “cognitive-perceptual” features without negative, disorganized, or impulsive features.^14^ Numerous studies of healthy samples have reported an association between schizotypy and neurological soft signs indicating subtle abnormalities of perceptual integration and motor coordination.^15–20^ In several studies, the association was most marked with negative schizotypy.^16,17,20^ Neurological soft signs are observed in a high proportion of cases of schizophrenia (see ref.^21^ for review). In particular, they are associated with disorganization and psychomotor poverty.^10^

Beta oscillations are rhythmic (13–30 Hz) changes in electrophysiological brain activity that are observable with electroencephalography (EEG) or magnetoencephalography (MEG). They play a major role in long-range communication in the brain^22^ and in particular, mediate feed-back influences through which higher brain centers/networks influence centers lower in the processing hierarchy.^23^ When a simple motor action is executed the amplitude of beta oscillations first drops below baseline, an effect known as event-related desynchronization (ERD), before rebounding above baseline. This post-movement beta rebound (PMBR) is localized to sensorimotor cortex.^24,25^

Evidence suggests that PMBR plays a role in visuomotor adaption, possibly related to maintenance or updating of the forward model that guides the execution of a motor act.^26^ Van Ede et al^27^ reported that tactile expectation modulates pre-stimulus beta-band oscillations in human sensorimotor cortex. Robson et al^28^ found that patients with schizophrenia exhibited a significant weakening of PMBR during a simple visuomotor task, compared to healthy controls, and furthermore that the degree of weakening of the PMBR was correlated with a composite score reflecting the severity of persisting symptoms and cognitive, occupational, and social disability. Whether similar deficits of PMBR are associated with schizotypal features is unknown.

We report an investigation of PMBR in healthy participants performing a visually cued movement task, similar to that employed by Robson et al.^28^ Participants completed a self-report questionnaire designed to measure features of schizotypal personality. We predicted that if subclinical schizotypy arises from similar neural mechanisms to schizophrenia, schizotypy in healthy individuals would be associated with reduced PMBR. Furthermore, we predicted that PMBR would be more strongly associated with those schizotypal features (disorganization; interpersonal) corresponding to the disorganization and/or negative symptoms of schizophrenia than with the cognitive-perceptual features corresponding more closely with the positive/reality distortion symptoms of schizophrenia.

## Methods

### Participants

All data were collected as part of the UK MEG Partnership (meguk.ac.uk, accessed 13 August 2018), a collaboration that aimed to collect normative data across a number of different tasks and scanner platforms. Healthy individuals (*N* = 166) were recruited at 2 sites—the Sir Peter Mansfield Imaging Centre, University of Nottingham (SPMIC), and Cardiff University Brain Research Imaging Centre (CUBRIC). Exclusion criteria were: any neurological condition; any mental illness in prior 5 years; any immediate family member with a diagnosis of schizophrenia; any MEG or magnetic resonance imaging (MRI) contraindications. All participants underwent identical MEG data acquisitions, and completed the Schizotypal Personality Questionnaire (SPQ).^29^ Participants with missing MEG or SPQ data, excessive head motion, poor task performance, or lack of motor peak (see Preliminary Beamforming below) were excluded from analyses, leaving 59 participants from SPMIC (26 males, age 19 to 62 y) and 53 participants from CUBRIC (16 males; age range 20 to 55 y). See supplementary S3 for demographics table and reasons for subject exclusion). The University of Nottingham Medical Ethics Board provided ethical approval for SPMIC and The Cardiff University School of Psychology Ethics Committee provided approval for CUBRIC.

### MEG Data Acquisition

Both sites used identical MEG parameters and instrumentation. Data were recorded using a 275-channel CTF system (MISL, Coquitlam, Canada) with third-order synthetic gradiometer configuration, sampling frequency = 1200 Hz. Participants were seated in the MEG system and fitted with 3 head position indicator coils, placed at naison, and left and right preauricular points. These enabled continuous recording of head position throughout the experiment. A 3-dimensional representation of each participant’s head shape (Polhemus Inc), was used to coregister MEG system geometry with their anatomical MRI.

### MRI Data Acquisition

SPMIC participants were scanned using a Philips 7T Achieva MRI scanner (Philips) and a phase sensitive inversion recovery (PSIR)^30^ sequence (field of view [FOV]: 240 × 216 × 160 mm^3^, 0.8 mm isotropic resolution). CUBRIC participants were scanned using a 3T GE system (General Electric) and a FSPGR (Fast SPoiled Gradient Recalled acquisition in the steady state) sequence (FOV: 256 × 192 × 168 mm^3^, 1 mm isotropic resolution). Scans were T_1_ weighted.

### Visuomotor Task

**Fig. 1.**
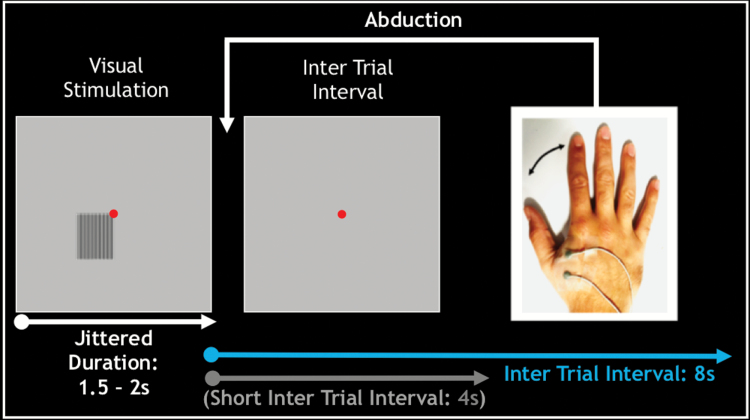
Schematic illustration of the visuomotor task. A high contrast grating was presented for a jittered duration of 1.5 to 2 s. At grating offset, participants made a single abduction of the right index finger. Grating offset was followed by an inter-trial-interval of 4 s or 8 s.

Participants completed a visuomotor task (figure 1). Two electrodes were positioned on the right dorsal interosseous and a ground reference positioned on the right lateral ulna to measure the electromyogram (EMG) response to finger abduction. Fifty trials incorporated a short inter trial interval (ITI) of 4 seconds, and a further 50 trials incorporated a long ITI of 8 seconds. As the PMBR response has been shown to last in excess of 6 seconds^24,31^ only the long ITI trials are analyzed here.

### Preliminary Beamforming

We used the SAM program from the CTF software package to locate voxels exhibiting a peak localization within the beta frequency band within a window 1–2 seconds post-abduction. Participants not exhibiting a peak in the left primary motor cortex were excluded from analyses (SPMIC *n* = 3, CUBRIC *n* = 6).

### Data Processing

**Fig. 2.**
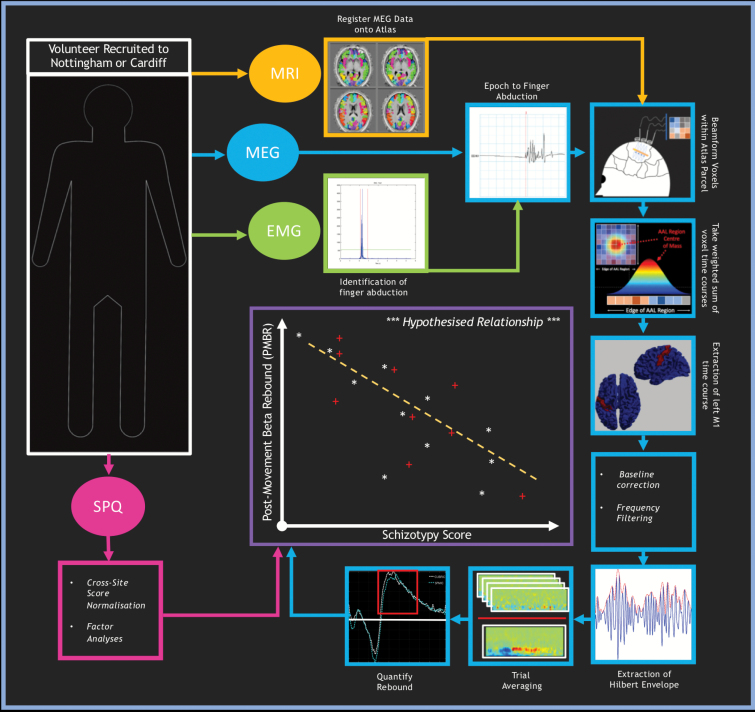
Overview of data processing. From each participant, we extracted 4 data streams (represented by ovals): magnetic resonance imaging (MRI), magnetoencephalography (MEG), electromyogram (EMG), and Schizotypal Personality Questionnaire (SPQ). We first used EMG to identify finger abductions. We subsequently epoched MEG data according to this marker. The automated anatomical labelling (AAL) atlas was then registered into anatomical space, via an anatomical MRI scan, and a beamformer used to derive an electrophysiological time course within each region. The time course corresponding to the left primary motor cortex was extracted; frequency filtered to the beta band, the Hilbert envelope derived, and trial averaged. A single value, capturing the amplitude of the beta rebound for each participant, was calculated based on the mean within a time window of interest. The SPQ data was normalized across sites and factor analyses performed. The SPQ and MEG streams were then combined to form our primary analysis assessing the relationship between post-movement beta rebound (PMBR) and schizotypy score (Note: correlation plot is illustrative only).

Figure 2 gives an overview of the data processing.

To identify the time of the finger abduction, the EMG data were rectified and thresholded using a previously described approach.^32^ Abductions greater than 3 SDs above the noise floor, within a time window spanning 100 ms < *t* < 900 ms relative to the offset of the visual stimulus were included. Data were then epoched such that the beginning of each trial (time-zero) was set to the start of the finger abduction. Following this, MEG data were inspected visually for artifacts by a single experimenter. Any trials containing excessive artifact, or where participants moved >5 mm from the starting position of the recording, were excluded. A mean of 8.7 trials were excluded per participant.

### Beamforming and Time Course Extraction

To assess PMBR, we employed brain parcellation and source space analysis.^33,34^ For each individual, the cortex was divided into 78 parcels according to the automated anatomical labelling atlas (AAL^35^); parcels were defined in standard (MNI) space and transformed to individual space.^36^ Each AAL region was divided into 4 mm cubic voxels and a beamformer-estimated time course of local electrical activity derived for each voxel (see supplementary S2.2 for beamformer parameters).^37–39^

### Time-Frequency Spectrograms

Time-frequency spectrograms (TFSs) averaged over trials were constructed by filtering broadband time courses into 31 overlapping frequency bands spanning 2–90 Hz. The amplitude envelope of the oscillations within each frequency band was computed as the absolute value of the analytic signal (generated using a Hilbert transform), and averaged over trials. To assess amplitude changes, a baseline window (the final second of each trial) was subtracted from the data.

### Spatial Signature of Beta Band Responses

To visualize the spatial distribution of beta modulation across the cortex, we averaged mid-beta band (16.5–25 Hz) amplitude envelope data in time windows centered over the ERD (−0.5 < *t* < 0.5 s) and the PMBR (1 < *t* < 2 s) peaks respectively, and plotted each as a function of AAL region.

### PMBR Quantification

To evaluate the PMBR amplitude, mid-beta band (16.5–25 Hz) amplitude envelope data from the left primary motor cortex were baseline corrected (using data from the final 1 s of each trial). A time window of interest (1–2 s post-abduction) was selected and amplitude values averaged across the duration of this window. This resulted in a single value characterizing the PMBR for each individual.

### Schizotypal Personality Scores

The SPQ^29^ is a 74-item self-report questionnaire designed to tap the 9 domains (supplementary S2.3) of SPD traits defined in the DSM-III-R.^40^ SPMIC participants self-completed an online 5-point Likert scale version of the questionnaire prior to arrival at the imaging center. CUBRIC participants completed a binary version of the same questionnaire in-person under the supervision of an experimenter. To align the 2 SPQ scoring methods, we normalized total scores using the means and SDs from normalization samples for each version (Likert and binary) collected by Wuthrich and Bates,^41^ and converted them to T-scores (population mean = 50; population SD =10).

### Factor Scores

We used confirmatory factor coefficients from Wuthrich and Bates’ Modified 3-Factor model^12^ to generate scores on each factor (“cognitive-perceptual,” “disorganization,” and “interpersonal”) from the domain scores.

### Statistical Analyses

All statistical analyses were performed in IBM Statistics SPSS 23. We first checked for differences between data acquired across the 2 sites: we ran bootstrapped independent *t*-tests (10000 samples; Bias-Corrected and Accelerated [BCa] standard errors and *P* values) for each SPQ measure, and also for age of participants. We conducted chi-square tests for significant differences in sex representation between sites, and tests for any association between sex or age and either PMBR or SPQ measures. We also checked for between-site differences in slope between SPQ measures and PMBR, by testing for significant site-by-SPQ interactions for each SPQ measure in turn, by means of a series of bootstrapped General Linear Models with PMBR as dependent variable. We then computed bootstrapped partial correlations between SPQ measures, and PMBR values, controlling for any effects of site, age, or sex. We also tested whether mean SPQ T-scores in our samples were significantly lower than the normalization samples.^41^

## Results

### Site Differences

**Fig. 3.**
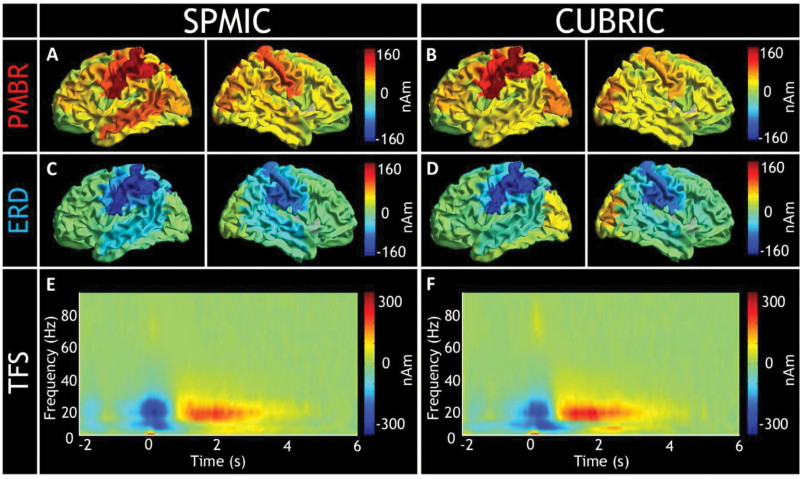
Assessing cross-site consistency. Sir Peter Mansfield Imaging Centre, University of Nottingham (SPMIC) results are presented in the panels on the left and Cardiff University Brain Research Imaging Centre (CUBRIC) results on the right. The top row (A and B) present amplitude change above (red) and below (blue) baseline, centered around the post-movement beta rebound (PMBR) (1–2 s). Note that in both cohorts, the region of maximal change is the left primary motor cortex. The second row also displays amplitude change from baseline, but centered around the event-related desynchronization (ERD; −0.5 to 0.5s). Note again that the location of absolute maximal power change is the left primary motor cortex. The final row shows group-averaged time-frequency spectrogram (TFS) generated from regions of interest in the left primary motor cortex. Note the similarity between the 2 cohorts in all cases.

In both cohorts, the greatest PMBR effects were found in the left primary motor region (figure 3A–D). TFSs from both cohorts clearly delineate the ERD and PMBR (figure 3E and 3F).

Bootstrapped independent *t*-tests indicated no significant differences between sites in PMBR or SPQ T-scores. However, factor scores on the interpersonal and cognitive-perceptual factors were significantly higher in the SPMIC sample, where the Likert version had been used (interpersonal: bootstrapped *P* < .001; cognitive-perceptual: bootstrapped *P* < .001). There were more female than male participants at both sites (SPMIC: 56% female; CUBRIC: 79% female), but the difference in proportions was not statistically significant, χ^2^(1) = 2.295, exact *P* = .171. However, the mean and variance of the age of the SPMIC participants was greater (mean = 39 y; SD = 11.9) than the CUBRIC participants (mean = 25 y; SD = 6.8), and this difference was statistically significant (bootstrapped *P* < .001, equal variances not assumed). We found no significant Site-by-SPQ interaction for either the SPQ T-scores, nor for any of the SPQ factor scores in predicting PMBR. We therefore treated site as a simple covariate for the remaining analyses, and included age and sex in additional analyses to check that results were not confounded by differences between the samples on these variables. There were no significant differences between male and female participants on PMBR values nor on any of the SPQ measures. Age was not a significant predictor of PMBR, nor was it significantly correlated with SPQ T-scores, nor with disorganization factor scores. However, age was significantly positively correlated with interpersonal factor scores (*r* = .274, bootstrapped *P* = .003) and cognitive-perceptual factor scores (*r* = .311, bootstrapped *P* = .001).

### Schizotypal Personality and PMBR

**Fig. 4.**
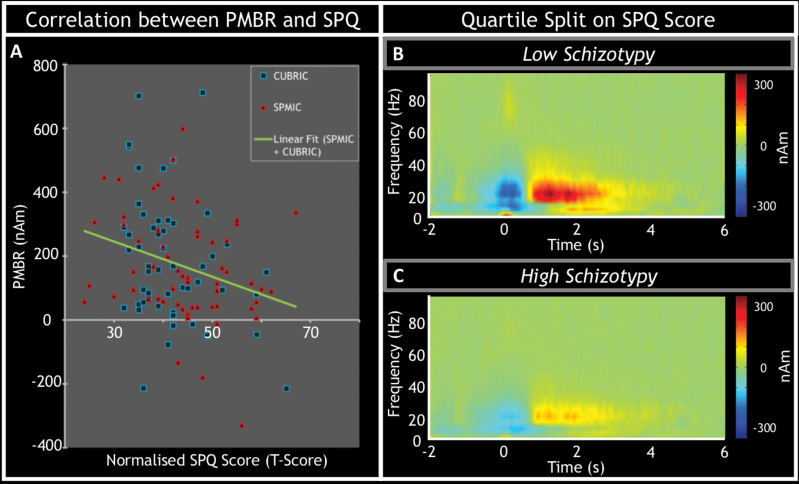
The relationship between post-movement beta rebound (PMBR) and schizotypal personality. (A) The correlation between the magnitude of the PMBR and normalized Schizotypal Personality Questionnaire (SPQ) T-scores. Red triangles indicate data from the Sir Peter Mansfield Imaging Centre, University of Nottingham (SPMIC) and blue squares represent that from Cardiff University Brain Research Imaging Centre (CUBRIC). The green line of best fit is fitted to both sets of data. The lessening of PMBR with increasing schizotypal personality is further visualized in panels (B) and (C) where the average time-frequency spectrograms from left primary motor cortex for the lowest and highest scoring participant quartiles (*n* = 28 in each) across groups. Notice the marked reduction in PMBR measured in participants with the highest schizotypy scores.

Figure 4A visualizes the relationship between SPQ score and PMBR amplitude. Here, data points in red represent subjects from the SPMIC cohort, while those in blue represent subjects from the CUBRIC cohort. Note the clear reduction in PMBR in those subjects with the highest SPQ score. Note that some participants had negative PMBR values. This was not because of a lack of a PMBR peak, but because the ERD was so great that the PMBR failed to return to above baseline levels.

Controlling for Site, SPQ T-scores were significantly negatively correlated with PMBR values (*r* = −.261, *df* = 109, bootstrapped *P* = .006). Of the SPQ factor scores, both interpersonal factor scores and disorganization factor scores were also significantly negatively correlated with PMBR (interpersonal: *r* = −.267, *df* = 109, bootstrapped *P* = .005; disorganization: *r* = −.282, *df* = 109, bootstrapped *P* = .003). There was no significant correlation between cognitive-perceptual factor scores and PMBR.

There were also significant positive correlations between each of the SPQ factors scores (interpersonal and cognitive-perceptual: *r* = .370, *df* = 109, bootstrapped *P* < .001; interpersonal and disorganization: *r* = .415, *df* = 109, bootstrapped *P* < .001; cognitive-perceptual and disorganization, *r* = .417, *df* = 109, bootstrapped *P* < .001). All these correlations remained statistically significant when age and sex were included in the models.

To investigate whether the variance in PMBR accounted by SPQ interpersonal and disorganization factor scores included variance shared between these factors, we computed partial correlations between each factor in turn while controlling for the other. After controlling for disorganization factor scores, there was only a marginally significant relationship between interpersonal factor scores and PMBR (*r* = −.171, *df* = 108, bootstrapped *P* = .071). However, after controlling for interpersonal factor scores, the partial correlation between disorganization and PMBR remained significant (*r* = −.195, *df* = 108, bootstrapped *P* = .041), though reduced in magnitude. This analysis indicates that the variance in PMBR accounted for by scores on these 2 factors includes variance that they share.

Finally, bootstrapped 1-sample *t*-tests indicated that SPQ T-scores were significantly lower than the normalization sample^41^ (population T-score mean = 50), bootstrapped *P* < .001 for both samples (SPMIC mean T-score = 44.4, SD = 9.8 and the CUBRIC mean was 41.5 (SD = 7.73).

## Discussion

Our results demonstrate a relationship between schizotypy and PMBR in which higher schizotypy scores are associated with reduced PMBR. Given our previous finding of reduced PMBR in schizophrenia,^28^ this finding supports the hypothesis of a continuum between schizotypy and schizophrenia, and suggests that diminution of PMBR reflects a neural mechanism that is expressed with increasing strength across a spectrum extending through the healthy population and via SPD into schizophrenia itself.

Our finding that this relationship is driven by variance shared by scores on the disorganization factor, and by the variance it shares with the interpersonal factor, and not with cognitive-perceptual factor scores, is consistent with our previous finding that PMBR magnitude decreased in proportion to the severity of a composite score representing persisting symptoms and functional impairment.^28^ In healthy schizotypy, Tabak and Weisman de Mamani^14^ found that individuals with high scores on the disorganization and interpersonal factors have lower levels of quality of life than those with only unusual experiences, indicating that scores on these factors are reflective of subjective experience of impairment.

In schizophrenia, disorganization and psychomotor poverty (which includes blunted affect and poverty of speech, both reflecting impaired interpersonal function) are associated with increased severity of cognitive, occupational and social dysfunction.^13,42^ In a longitudinal study of a general population sample of adolescents and young adults, Dominguez et al^43^ found that expression of negative/disorganized symptoms predicted psychotic experiences and subsequent clinical psychosis. Similarly, in a longitudinal study of adolescents consulting for nonpsychotic difficulties, Debbané et al^44^ found that disorganization features mediate the relationships between the negative and positive dimensions of schizotypy within and across evaluations. They conclude that the relationship between disorganization features and positive schizotypy may play a central role in establishing risk for psychosis during adolescence. Similarly, in a longitudinal study of ultra-high-risk (UHR) adolescents, scores on the disorganization subscale of the Structured Interview for the Assessment of Prodromal Syndromes^45^ at baseline predicted severity of impaired function assessed using the Global Assessment of Function 6 years later.^46^

As the healthy participants in our study were not receiving antipsychotic medication, the diminution of PMBR cannot be attributed to medication. This, in turn, reduces the likelihood that the findings of Robson et al^28^of reduced PMBR in schizophrenia were an artifact of anti-psychotic medication. Rather, our findings suggest continuity between a neural mechanism associated with schizotypy in the healthy population and the neural mechanism underlying similar features observed in schizophrenia.

While the neural mechanism that generates PMBR remains a topic of debate, GABAergic transmission is implicated. For example, using Magnetic Resonance Spectroscopy, Gaetz et al^47^ observed a significant linear relationship between GABA concentration in primary motor cortex and PMBR power. Furthermore, PMBR is associated with the process of maintaining or adapting the brain’s internal model that controls movements based on a prediction of the consequences of those movements. Variation in the magnitude of PMBR is greater when the discrepancy between the actual consequence of an action and the intended consequence is small, and furthermore this effect is increased if the prior performance history indicates that errors provide information that is useful for updating the brain’s internal model.^48^ Cao and Hu^26^ propose that high beta rebound is associated with the process of actively maintaining the current forward model that guides movement. This interpretation would be consistent with Asai et al’s^49^ finding that schizotypal personality traits correlated with deficits in prediction of one’s own reaching movements under a no-visual-feedback condition, and suggest that our finding of the relationship of reduced PMBR with higher scores on disorganization and interpersonal factors of schizotypy may be related to impairment of a neural mechanism that plays a role in the internal regulation of behavior.

We should note that in the Robson et al^28^ study, participants pressed a button repeatedly during the visual stimulus (2 s), whereas here, participants made a single lateral finger abduction at the offset of the stimulus. Both protocols elicited PMBR, but the protocol in the present study enabled greater precision in isolating the timing of motor execution.

Future research should include assessment of PMBR in individuals at clinical high risk and in early phase cases. Notably, in a MEG study of a small sample of children and adolescents with early onset schizophrenia, Wilson et al^50^ reported multiple electrophysiological abnormities during finger movements, including reduced PMBR in diverse brain regions. As the effects reported here might be mediated by other personality factors, future studies should also include comprehensive psychometric assessments to allow investigation of possible mediators, such as cognitive factors or clinical features, such as anxiety or depression.

The finding that diminution of PMBR, previously reported in schizophrenia, is correlated with the severity of schizotypal features across the range observed in the general population, supports the hypothesis that at least some aspects of schizophrenia lie at the extreme end of a normal personality variant. Moreover, the finding that decreased PMBR is associated with experiences similar in character to those associated with occupational and social dysfunction in schizophrenia suggests that the mechanism of these symptoms in schizophrenia is not categorically distinct from that responsible for similar features in the healthy population.

## Funding

This work was supported by the Medical Research Council (grant numbers MR/K005464/1, MR/M006301/1, MR/J01186X/1, MR/K501086). The fourth grant was an MRC Doctoral Training Grant supporting B.A.E.H., L.M., and B.C.R.

## Supplementary Material

sby117_suppl_Supplementary_Material
