# Supplementary material for: Attenuated Post-Movement Beta Rebound Associated With Schizotypal Features in Healthy People
Source: Schizophr Bull. 2018 Sep 18;45(4):883–91. doi: 10.1093/schbul/sby117 (PMC6581139; doi:10.1093/schbul/sby117)
Supplement: sby117_suppl_Supplementary_Material [file sby117_suppl_supplementary_material.doc]

# **Supplementary Information:**

Our supplementary information is split into three sections. S1 presents further analyses assessing for any confounding influences that may detract from our primary results. Due to space constraints in the main text, we also present S2 - Extended methodology. Finally, section S3 comprises two tables with demographic information with statistical comparisons between the samples from SPMIC and CUBRIC, and detailed information on subject exclusion.

## **S1 – Assessing the influence of potentially confounding factors**

## **S1.1 Correlation between Schizotypy Score and Baseline Power:**

As we are assessing PMBR as a change from baseline, it is conceivable that the observed effects could be due to baseline differences between high and low schizotypal personalities. To assess this potential confound, we used Pearson correlation to test for any significant relationship between schizotypy score and mean baseline power (averaged over trials) within the same mid-beta frequency band. The results of this comparison are presented in Figure S1. Note that there were no significant or trending correlations between baseline power and schizotypy score in either cohort.

Figure S1: Relationship between baseline power and schizotypy score. Note in both data from Nottingham (SPMIC) and Cardiff (CUBRIC) there is no significant or trending relationship between baseline power and schizotypy score.

## **S1.2 Correlation between Schizotypy Score and Number of Trials Removed:**

To ensure our results are not driven by signal to noise ratio (SNR) differences between participants at either end of the schizotypy spectrum, we assessed if there was any relationship between the number of trials removed (due to artefact, head movement, or poor task performance) and schizotypy score. We assessed this with Person correlations and found there to be no significant relationships in either the SPMIC or CUBRIC samples. These results are presented in Figure S2.


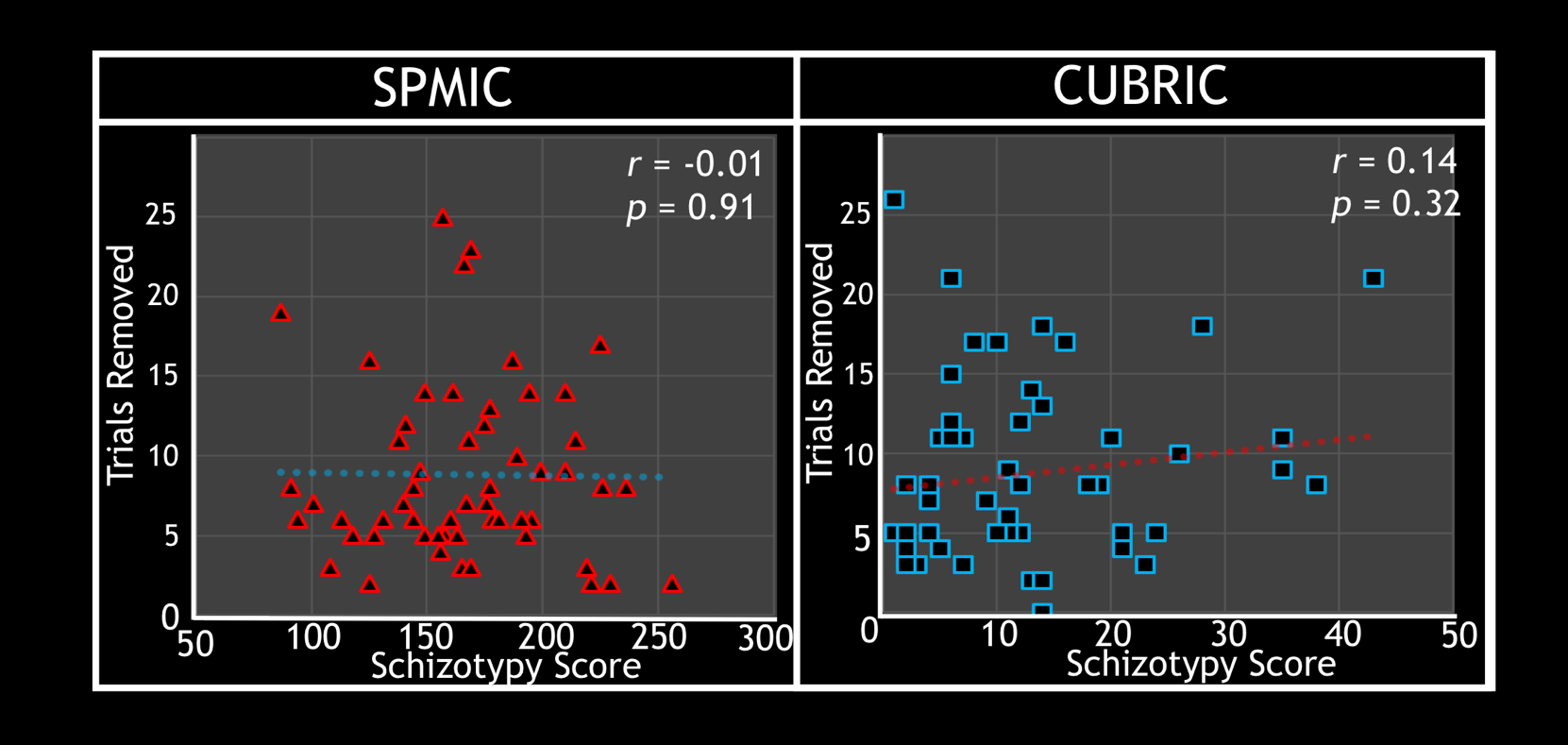


*Figure S2: Relationship between Schizotypy Score and the number of trials of the visuo-motor task removed, plotted separately for participants recruited to Nottingham (SPMIC) and Cardiff (CUBRIC). In both cases, Pearson correlation revealed there to be no significant relationship (p>0.05).*

## **S1.3 Potential Relations to Schizotypy Score:**

To ensure that our measured variables were not mediating schizotypy scores (other than PMBR) we analysed, separately for each site, for relations between schizotypy score and response/abduction time, age, and sex using Pearson correlation or, when analysing sex, a Wilcoxon Signed Rank Test. We found there to be no significant associations in either cohort (Figure S3).

*Figure S3: Relationship between Schizotypy Score and (A) Response/Abduction time, (B) Age, and (C) Sex. We found there to be no significant relations in any test.*

## **S1.4 Potential Relations to Post Movement Beta Rebound:**

Finally, To ensure that our measured variables (other than schizotypy score) were not related to PMBR we analysed for relations between PMBR and response/abduction time, age, and sex using Pearson correlation or a Wilcoxon Signed Rank Test. Again we found there to be no significant associations.

*Figure S4: Relationship between PMBR and (A) Response/Abduction time, (B) Age, and (C) Sex. We found there to be no significant relations in any test.*

S2 – Extended Methodology:

## **S2.1 Visuo-motor Task**

Participants were seated in the MEG system and completed a visuomotor task. Each trial began with the presentation of a high contrast visual grating (maximum contrast, 3-cycles per degree, vertical square-wave grating covering approximately 4x4 degrees of the lower left visual field) presented on a grey mean-luminance background for a jittered duration of 1.5 to 2 seconds. A red fixation point was positioned at the upper right corner of the grating in the centre of the screen and was visible constantly. Participants were instructed to fixate on this point and to make a single abduction of their right index finger at the offset of the visual grating. Visual presentation was made via projection, through a waveguide in the shielded room, onto a screen positioned ~40cm in front of the participant’s eyes. To record the precise timing of finger abductions, two electrodes were positioned on the right dorsal interosseous and a ground reference positioned on the right lateral ulna. Electromyogram (EMG) data were recorded simultaneously with the MEG to facilitate later processing. To increase the flexibility of the data, two inter-trial-interval (ITIs) durations were used. Participants completed 50 trials incorporating a short ITI of 4s, and a further 50 trials of a long ITI, lasting 8s. Trials were presented in a random order. In the present study we were interested only in the PMBR; as this response has been shown to last in excess of 6s^1^, *only* the long ITI trials were analysed here. (Previous work^2^ has shown that too short an ITI obfuscates the magnitude of the PMBR).

## **S2.2 Beamforming and timecourse extraction:**

To assess PMBR, we employed a brain parcellation and a source space analysis. For each individual, the cortex was divided into 78 parcels according to the automated anatomical labelling atlas (AAL)^3^; parcels were defined in standard (MNI) space and transformed to individual space using FLIRT in FSL^4^. Each AAL region was divided into 4mm cubic voxels and a beamformer-estimated timecourse of local electrical activity derived for each voxel^5–7^. For beamforming, we computed a data covariance matrix in a 1-150Hz frequency window and a time window spanning the entire experiment^8^. Regularisation was applied using the Tikhonov method with a regularisation value equal to 5% of the maximum eigenvalue of the unregularized matrix. The forward solution employed a dipole approximation^9^ and a multiple local sphere head model^10^. To generate a single representative timecourse for each AAL region, individual voxel timecourses were combined in a weighted sum, with weightings defined by a 3D Gaussian function (full width at half maximum of 17mm). The centre of the Gaussian function was at the centre of mass of the AAL region, meaning voxels lying on regional boundaries were down-weighted^11^.

## **S2.3 Schizotypal personality scores:**

The SPQ^12^ is a 74-item self-report questionnaire. It was designed to tap the nine domains of schizotypal personality disorder traits defined in the DSM-III-R^13^; specifically 1) ideas of Reference (9 items); 2) Excessive Social Anxiety (8 items); 3) Odd Beliefs or Magical Thinking (7 items); 4) Unusual Perceptual Experiences (9 items); 5) Odd or Eccentric Behaviour (7 items); 6) No Close Friends (8 items); 7) Odd Speech (9 items); 8) Constricted Affect (9 items); 9) Suspiciousness (8 items). Participants recruited to the SPMIC completed a digitised version of the questionnaire through Bristol Online Surveys (bos.ac.uk) prior to arrival at the imaging centre. They were asked to rate the extent to which each SPQ item applied to them using a five-point Likert scale. Responses were scored as follows: strongly disagree = 0, disagree = 1, neutral = 2, agree = 3, strongly agree = 4. Participants recruited to CUBRIC completed the same questionnaire in-person under the supervision of an experimenter. These participants were simply asked to respond “Yes” (score=1), or “No” (score=0) to each item. To make the two sets of SPQ scores comparable, we normalised total scores using the means and standard deviations from normalisation samples for each version (Likert and binary) collected by Wuthrich and Bates^14^, and converted them to T scores (scores with a population mean of 50 and population SD of 10).

### **S2.4 Factor scores:**

As noted in our introduction, a three factor structure (similar to that observed in overt schizophrenia) has emerged from factor analyses of schizotypy^15,16^, the three factors being “Unusual Experience”, “Disorganization” and “Negative”. Here, we used confirmatory factor coefficients from Wuthrich and Bates’^17^ Modified Three-Factor model to generate scores on each factor from the domain scores. In the Wuthrich and Bates model, a Disorganization factor loads on *Odd or Eccentric Behaviour* and *Odd Speech*; an Interpersonal factor loads on *Excessive Social Anxiety*, *No Close Friends,* and *Constricted Affect*; and a Cognitive-Perceptual factor loads on *Ideas of Reference*, *Odd Beliefs or Magical Thinking*, and *Unusual Perceptual Experiences*. *Suspiciousness* has loadings on both the Interpersonal and Cognitive-Perceptual factors. To give factor scores with similar scales across the two recording sites, we first divided the Nottingham Likert question scores by 4, so that for both binary and Likert versions, the maximum score on each question was 1 and the minimum was 0.

### **S2.5 Participant screening**

Participants completed the UK MEG Partnership online self-report screening form before being invited to take part in the study. Two of the most relevant items are pasted below. Participants who answered ‘Yes’ to the mental illness item were excluded if the illness and any associated treatment had occurred within the past 5 years. Participants responding ‘Yes’ to the second question were excluded from the study, regardless of how much time had passed since their last ‘episode’.

- Have you ever suffered from a mental illness such as depression or schizophrenia?
  - (if Yes) What was your diagnosis?
  - (if Yes) When was your diagnosis?
  - (if Yes) What treatment are you currently receiving
- Have you ever suffered from a neurological condition (e.g. Stroke, Traumatic Brain Injury or Epilepsy)?
  - (if Yes) What was your diagnosis?
  - (if Yes) When was your diagnosis?
  - (if Yes) What treatment are you currently receiving

Participants also completed a questionnaire on the day of scanning that questioned medication, caffeine and alcohol intake for 24 hours prior to the scan. One subject was excluded as they had been prescribed anti-depressant medications between the initial screening and the scanning day.

## **S3 – Sample Demographics & Reasons for Participant Exclusion**

### **S3.1 Sample Demographics:**

The table below describes the mean, standard deviation, and range for age, schizotypy score, number of trials, PMBR value, and response/abduction time separated by site and sex, and also calculated on the entire cohort.

*Table S1 – Demographic information and statistics. As expected, age significantly differed between sites. We also found schizotypy t-score to marginally differ between sites.*

### **S3.2 Reasons for Subject Exclusion:**

The table below presents the reasons for subject exclusion, separated by site, and included demographic data for those subjects (where available).

*Supplementary Table 2: Reasons for subject exclusion and associated demographic information (where available).*

To elaborate on the less self-explanatory reasons noted above:

- Head Movement: Participant exceeded the 5mm motion threshold for a significant portion of the task (> 25/50% of trials) and had insufficient data remaining for inclusion in the analyses.
- Technical Issue: Scanning malfunction, either with MEG, Stimulus Computer, or detachment of EMG electrodes.
- No Motor Peak: In our preliminary analysis, participants without a peak voxel in the left primary motor cortex were excluded from analyses.
- Task Failure: Participant failed to make responses within the response window (either too quick or too short) in > 50% of trials.
- Ocular Interference: One participant in the Nottingham cohort blinked with such vigour as to cause highly disruptive artefacts that could not be removed with post-processing.
- Antidepressant Medication: One participant in the Nottingham cohort failed to disclose that they had recently started taking antidepressant medication until after the scanning session.

**Supplementary Information References:**

1. Pfurtscheller G, Lopes da Silva FH. Event-related EEG/MEG synchronization and desynchronization: basic principles. *Clin Neurophysiol*. 1999;110(11):1842-1857. doi:10.1016/S1388-2457(99)00141-8

2. Fry A, Mullinger KJ, O’Neill GC, et al. Modulation of post‐movement beta rebound by contraction force and rate of force development. *Hum Brain Mapp*. 2016;37(7):2493-2511. doi:10.1002/hbm.23189

3. Tzourio-Mazoyer N, Landeau B, Papathanassiou D, et al. Automated anatomical labeling of activations in SPM using a macroscopic anatomical parcellation of the MNI MRI single-subject brain. *NeuroImage*. 2002;15(1):273-289. doi:10.1006/nimg.2001.0978

4. Smith SM, Jenkinson M, Woolrich MW, et al. Advances in functional and structural MR image analysis and implementation as FSL. *NeuroImage*. 2004;23 Suppl 1:S208-219. doi:10.1016/j.neuroimage.2004.07.051

5. Van Drongelen W, Yuchtman M, Van Veen BD, Huffelen AC van. A spatial filtering technique to detect and localize multiple sources in the brain. *Brain Topogr*. 1996;9(1):39-49. doi:10.1007/BF01191641

6. Van Veen BD, van Drongelen W, Yuchtman M, Suzuki A. Localization of brain electrical activity via linearly constrained minimum variance spatial filtering. *IEEE Trans Biomed Eng*. 1997;44(9):867-880. doi:10.1109/10.623056

7. Robinson SE, Vrba J. Functional neuroimaging by synthetic aperture magnetometry (SAM). *Recent Adavances Biomagn Ed Yoshimoto T Kotani M Kuriki Karibe HNakasato N Univ Press Sendai Jpn*. 1998:302-305.

8. Brookes MJ, Vrba J, Robinson SE, et al. Optimising experimental design for MEG beamformer imaging. *NeuroImage*. 2008;39(4):1788-1802. doi:10.1016/j.neuroimage.2007.09.050

9. Sarvas J. Basic mathematical and electromagnetic concepts of the biomagnetic inverse problem. *Phys Med Biol*. 1987;32(1):11. doi:10.1088/0031-9155/32/1/004

10. Huang MX, Mosher JC, Leahy RM. A sensor-weighted overlapping-sphere head model and exhaustive head model comparison for MEG. *Phys Med Biol*. 1999;44(2):423-440.

11. Brookes MJ, Tewarie PK, Hunt BAE, et al. A multi-layer network approach to MEG connectivity analysis. *NeuroImage*. 2016;132:425-438. doi:10.1016/j.neuroimage.2016.02.045

12. Raine A. The SPQ: A Scale for the Assessment of Schizotypal Personality Based on DSM-III-R Criteria. *Schizophr Bull*. 1991;17(4):555-564. doi:10.1093/schbul/17.4.555

13. American Psychiatric Association. *Diagnostic and Statistical Manual of Mental Health Disorders (DSM-III-R)*. American Psychiatric Association; 1987.

14. Wuthrich V, Bates TC. Reliability and validity of two Likert versions of the Schizotypal Personality Questionnaire (SPQ). *Personal Individ Differ*. 2005;38(7):1543-1548. doi:10.1016/j.paid.2004.09.017

15. Bentall RP, Claridge GS, Slade PD. The multidimensional nature of schizotypal traits: a factor analytic study with normal subjects. *Br J Clin Psychol*. 1989;28 ( Pt 4):363-375.

16. Wuthrich VM, Bates TC. Confirmatory factor analysis of the three-factor structure of the schizotypal personality questionnaire and Chapman schizotypy scales. *J Pers Assess*. 2006;87(3):292-304. doi:10.1207/s15327752jpa8703_10

17. Wuthrich VM, Bates TC. Confirmatory Factor Analysis of the Three-Factor Structure of the Schizotypal Personality Questionnaire and Chapman Schizotypy Scales. *J Pers Assess*. 2006;87:292-304. doi:10.1207/s15327752jpa8703_10
